# Correction: On people’s perceptions of climate change and its impacts in a hotspot of global warming

**DOI:** 10.1371/journal.pone.0323273

**Published:** 2025-04-30

**Authors:** The PLOS One Staff

The authors, Meghnath Dhimal and Ruth Müller, should have been noted as shared last co-authors not shared first co-authors on this work.

The first two authors, Parbati Phuyal and Isabelle Marie Kramer should be noted as shared first co-authors on this work.

The publisher apologizes for the errors.

## References

[1] Phuyal P, Kramer IM, Kadel I, Wouters E, Magdeburg A, Groneberg DA, et al. On people’s perceptions of climate change and its impacts in a hotspot of global warming. PLoS One. 2025;20(2):e0317786. doi: 10.1371/journal.pone.0317786 39946319 PMC11825050

