# Supplementary figures and images for: On people’s perceptions of climate change and its impacts in a hotspot of global warming
Source: PLoS One. 2025 Feb 13;20(2):e0317786. doi: 10.1371/journal.pone.0317786 (PMC11825050; doi:10.1371/journal.pone.0317786)

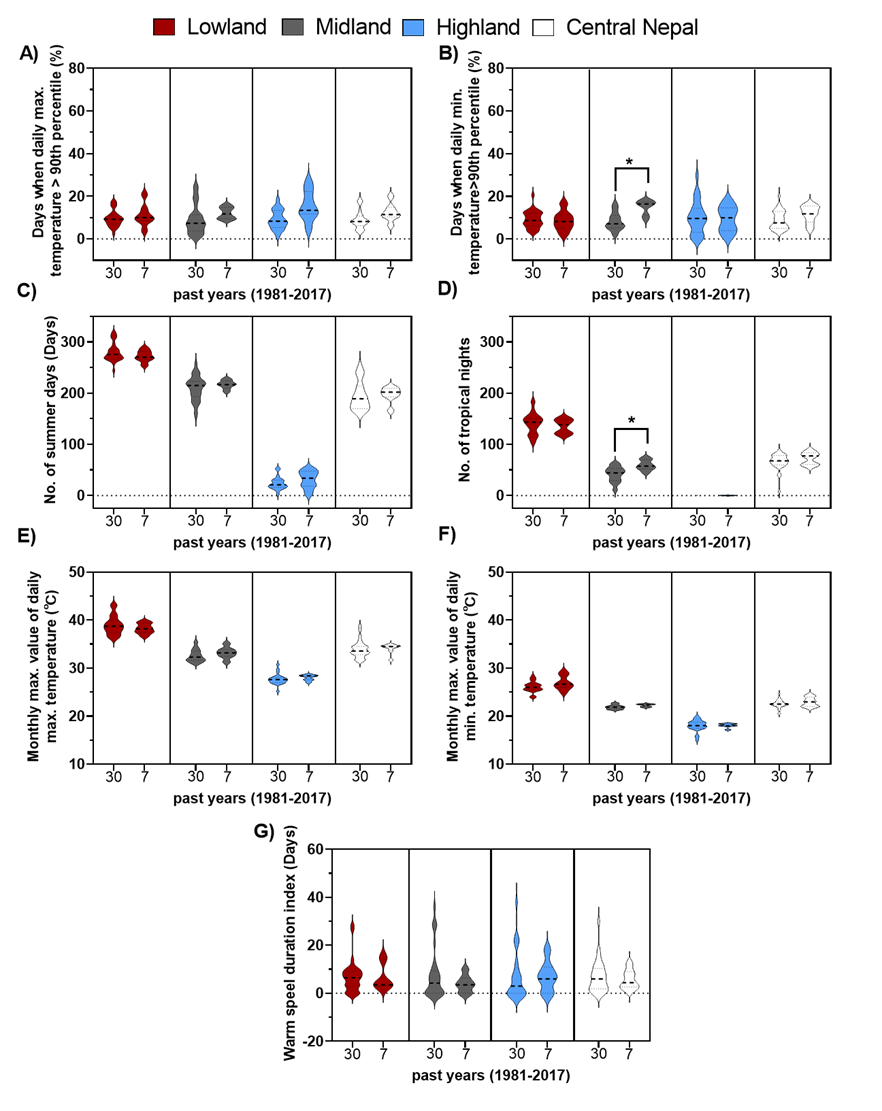

Supplement: S1 Fig — Results are given for the Lowland, Midland and Highland and also across the altitudinal gradient in Central Nepal during a past 30 year’s period (1981–2010) vs. past 7 years (2011–2017). Climatic indices: A) TX90P- warm days, B) TN90P- warm nights, C) SU-summer days, D) TR-Tropical nights, E) TXx- warmest day F) TNx-warmest night and G) WSDI- warm spell duration indicator. Significant climate indicators (Mann-Whitney U-test) in the 30 years (1981–2010) vs. 7 years (2011–2017) comparison after FDR correction are marked with an asterisk. (TIF) [file pone.0317786.s001.tif]

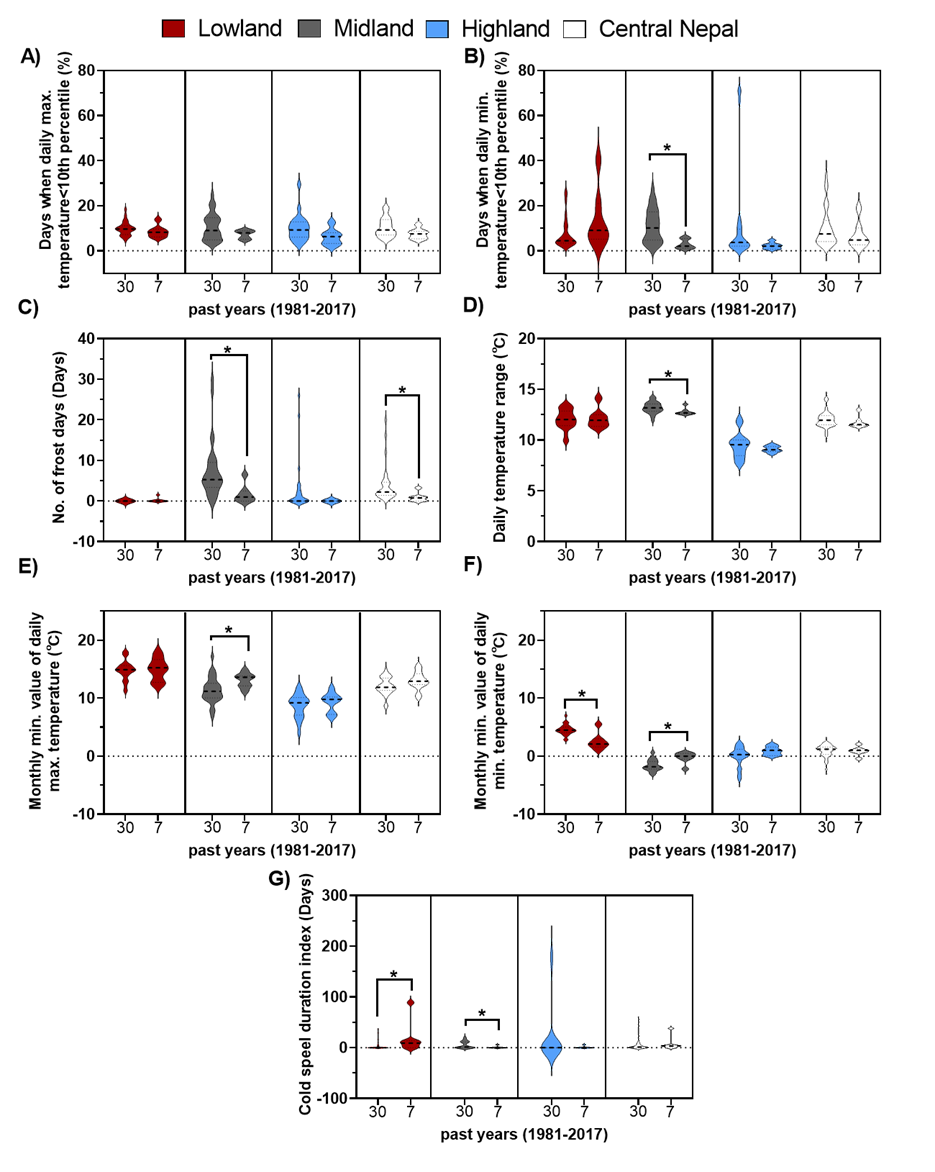

Supplement: S2 Fig — Results are given for the Lowland, Midland, Highland and also across the altitudinal gradient in Central Nepal during a past 30 year- period (1981–2010) vs. past 7 years (2011–2017). Climatic indices: A) TX10P- cool days, B) TN10P- cool nights, C) FD- frost days, D) DTR- daily temperature range, E) TXn- coldest day F) TNn- coldest night and G) CSDI- cold spell duration indicator. Significant climate indicators (Mann-Whitney U-test) in the 30 years (1981–2010) vs. 7 years (2011–2017) comparison after FDR correction are marked with an asterisk. (TIF) [file pone.0317786.s002.tif]

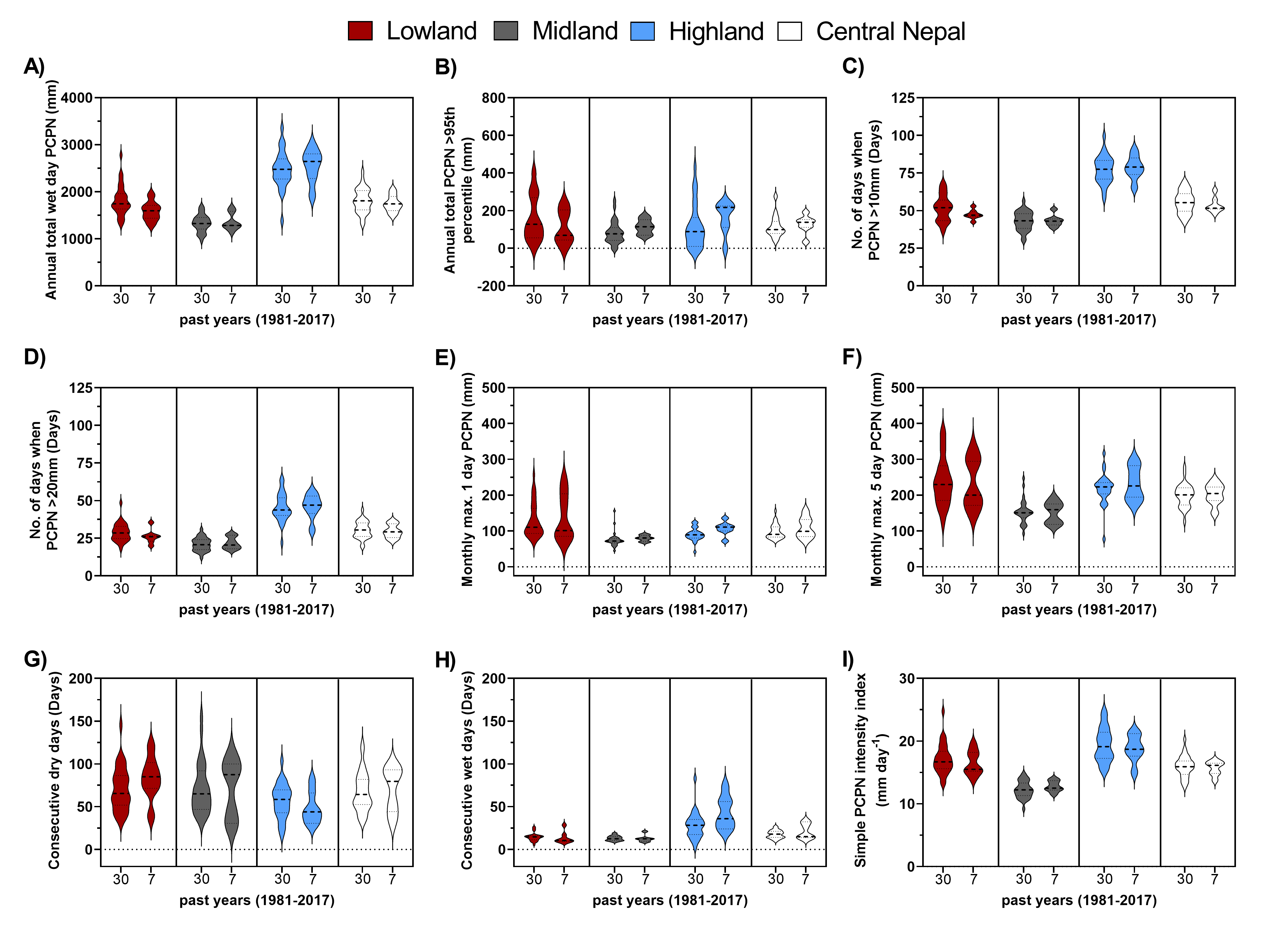

Supplement: S3 Fig — Results are given for the Lowland, Midland and Highland and also across the altitudinal gradient in Central Nepal during a past 30 years-period (1981–2010) vs. the past 7 years (2011–2017). Climatic indices: A) PCRPTOT- annual total wet-day precipitation, B) R95PTOT- precipitation on very wet days, C) R10MM- number of heavy precipitation days, D) R20MM- number of very heavy precipitation days, E) RX1Day PCPN- monthly max 1 day precipitation amount, F) RX5Day PCPN- monthly max. 5 day precipitation amount, G) CDD- consecutive wet days, H) CWD- consecutive dry days, and I) SDII- simple daily precipitation intensity index. (TIF) [file pone.0317786.s003.tif]

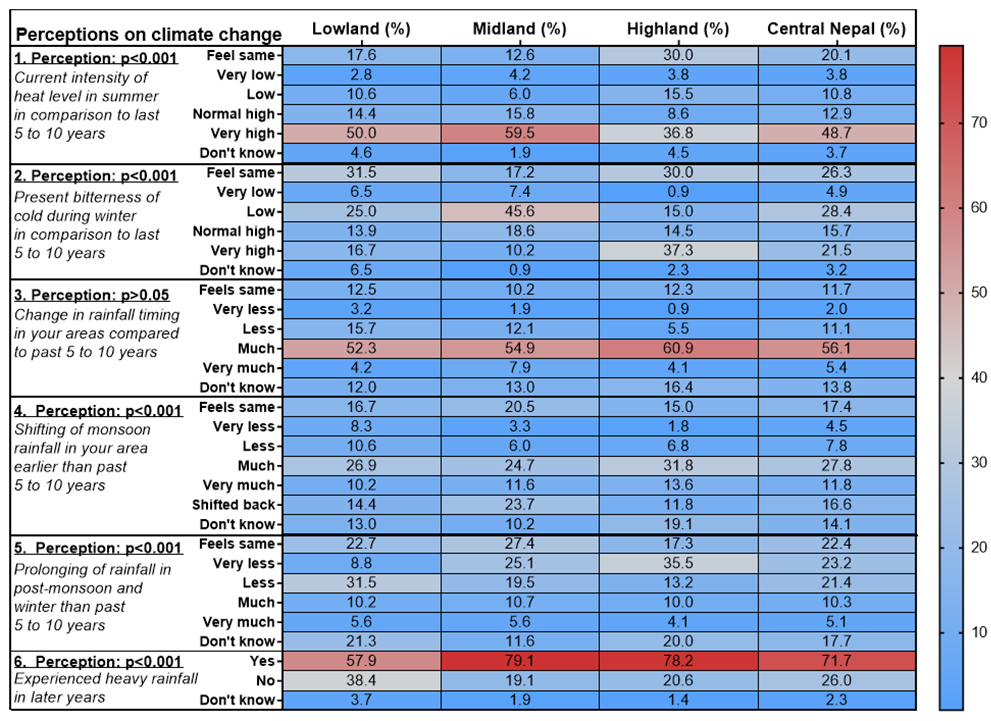

Supplement: S4 Fig — Heat map of the perceptions P1-P6 on climate change and climatic variability (% participants; p-value from the Chi-square test) in accordance with the altitudinal residence of the participants in the Lowland, Midland and Highland or their general residence in Central Nepal. (TIF) [file pone.0317786.s004.tif]

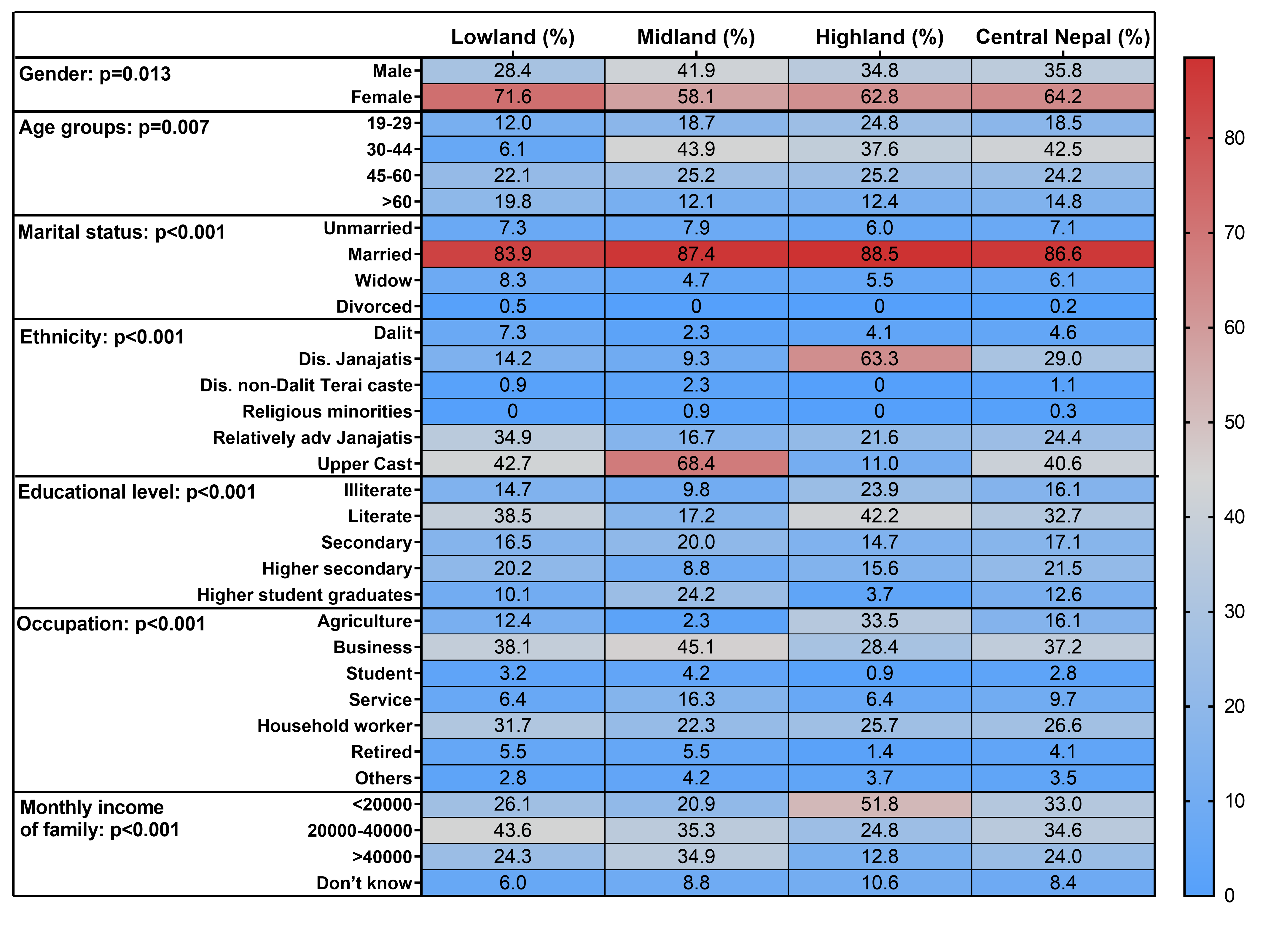

Supplement: S5 Fig — Heat map of the socio-demographic characteristics (% participants; p-value from the Chi-square test) in accordance with the altitudinal residence of the participants in the Lowland, Midland and Highland or their general residence in Central Nepal. (TIF) [file pone.0317786.s005.tif]

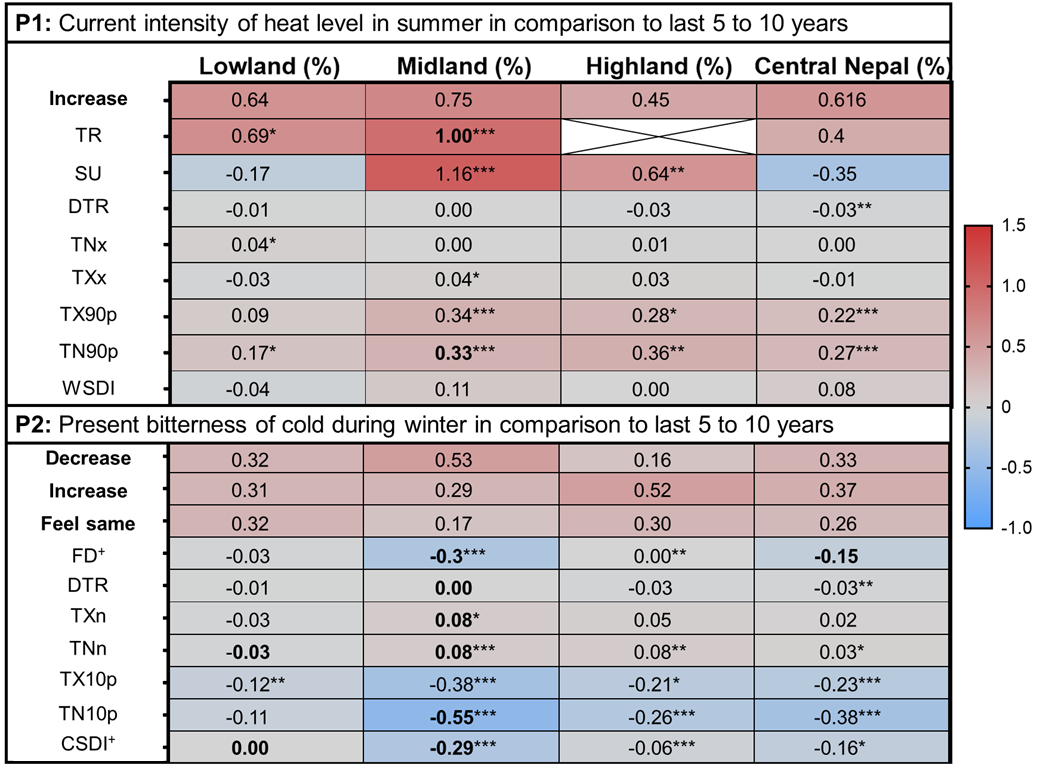

Supplement: S6 Fig — Heat map of the people’s perceptions of P1 and P2 (given as fractions of the total answers) and the trends (Sen’ slope) over the last 37 years (1981–2017) of the heat or cold-related annual climate extreme indices (Table 1) in accordance with the residence of the participants in the Lowland, Midland and Highland or their general residence in Central Nepal. Significant climate indicators from the trend analyis ((Mann- Kendall test) over the last 37 years (1981–2017) are marked with an asterisk (* p < 0.05, ** p < 0.01, *** p < 0.0001). Significant climate indicators (Mann-Whitney U-test) in the 30 years (1981–2010) vs. 7 years (2011–2017) comparison after FDR correction are marked in bold. In the Highland no tropical nights (TR) were recorded. (TIF) [file pone.0317786.s006.tif]

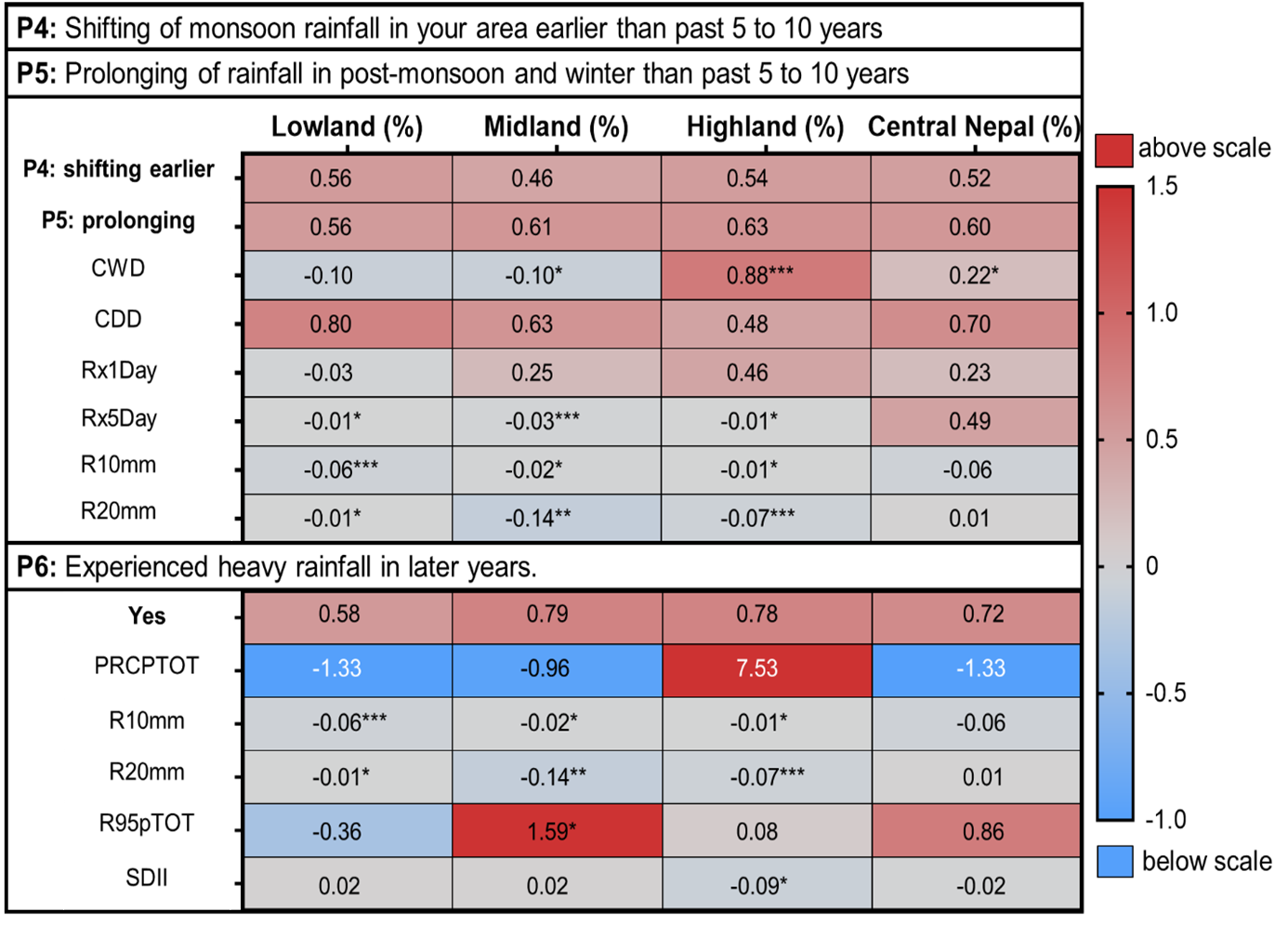

Supplement: S7 Fig — Heat map of perceptions P4 –P6 (given as fractions of the total answers) and the trend (Sen’s slope) over the last 37 years (1981–2017) of annual precipitation-related climate extreme indices (Table 1) in accordance with the residence of participants in the Lowland, Midland and Highland or their general residence in Central Nepal. Significant indicators from the trend analyis (Mann- Kendall test) over the last 37 years (1981–2017) are marked with a asterisk(* p < 0.05, ** p < 0.01, *** p < 0.0001). Significant climate indicators (Mann-Whitney U-test) in the 30 years (1981–2010) vs. 7 years (2011–2017) comparison after FDR correction are marked in bold. (TIF) [file pone.0317786.s007.tif]

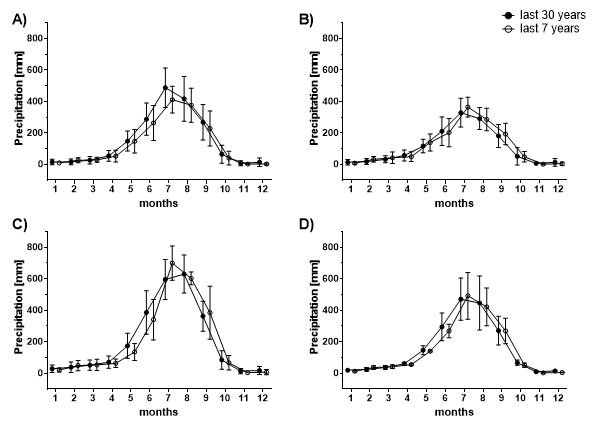

Supplement: S8 Fig — Precipitation is shown for the regions A) Lowland, B) Midland, C) Highland, and D) Central Nepal. (TIF) [file pone.0317786.s008.tif]

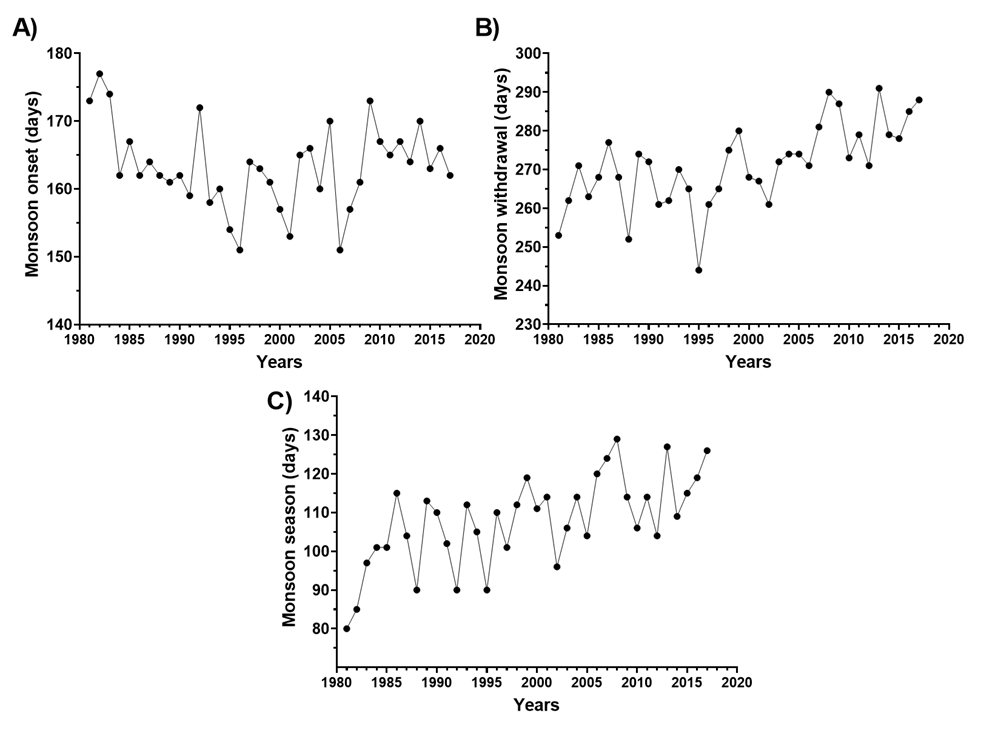

Supplement: S9 Fig — Monsoon onset (A), monsoon withdrawal (B) and the monsoon period (C; in days) from 1981–2017 in Central Nepal. Data of the year 1980 is missing. Figures are adjusted using data/figures from the Department of Hydrology and Meteorology, Kathmandu Nepal (http://dhm.gov.np/download/). The 37-year trend analysis: Sen’s slope: A) -0.02 (p = not significant), B) 0.65 (p<0.0001), C) 0.71 (p<0.0001). 30 years vs. 7 years analysis: Mann-Whitney test: A) p = not significant, B) p<0.01, C) p<0.05). (TIF) [file pone.0317786.s009.tif]
