# Supplementary material for: On people’s perceptions of climate change and its impacts in a hotspot of global warming
Source: PLoS One. 2025 Feb 13;20(2):e0317786. doi: 10.1371/journal.pone.0317786 (PMC11825050; doi:10.1371/journal.pone.0317786)
Supplement: S2 Table — (DOCX) [file pone.0317786.s011.docx]

**S2 Table.** **Description of weather stations per study region.**

|  | **Lowland** | | **Midland** | | **Highland** | |
| --- | --- | --- | --- | --- | --- | --- |
| **District** | Chitwan | Dhading | Kathmandu | Lalitpur | Rasuwa | Kakani |
| **City** | Rampur | Dhunebesi | Kathmandu airport | Khumaltar | Dhunche | Kakani |
| **Station number** | 902 | 1038 | 1030 | 1029 | 1055 | 1007 |
| **Distance to social data collection** | 19.6 km | 62.3 km | 5 km | 5.9 km | 3.15 km | 2.8 km |
| **Altitude [m]** | 256 | 988 | 1337 | 1350 | 1982 | 2060 |
