# Supplementary material for: On people’s perceptions of climate change and its impacts in a hotspot of global warming
Source: PLoS One. 2025 Feb 13;20(2):e0317786. doi: 10.1371/journal.pone.0317786 (PMC11825050; doi:10.1371/journal.pone.0317786)
Supplement: S3 Table — Given per study region (Lowland, Midland and Highland) and per 30-year baseline (1981–2010) or for the last 7 years (2011–2017). (DOCX) [file pone.0317786.s012.docx]

**S3 Table.** **Amount of missing values** **of climate extreme indices** (%). Given per study region (Lowland, Midland and Highland) and per 30-year baseline (1981-2010) or for the last 7 years (2011-2017).

|  | **Lowland** | | **Midland** | | **Highland** | |
| --- | --- | --- | --- | --- | --- | --- |
|  | **30 years** | **7 years** | **30 years** | **7 years** | **30 years** | **7 years** |
| **Temperature indicators** | | | | | | |
| TX90P | 0.0 | 0.0 | 0.0 | 0.0 | 26.7 | 0.0 |
| TN90P | 3.3 | 0.0 | 0.0 | 0.0 | 13.3 | 0.0 |
| TX10P | 0.0 | 0.0 | 0.0 | 0.0 | 26.7 | 0.0 |
| TN10P | 3.3 | 0.0 | 0.0 | 0.0 | 13.3 | 0.0 |
| SU | 0.0 | 0.0 | 0.0 | 0.0 | 21.6 | 0.0 |
| TR | 3.3 | 0.0 | 0.0 | 0.0 | 13.3 | 0.0 |
| FD | 3.3 | 0.0 | 0.0 | 0.0 | 13.3 | 0.0 |
| TNx | 3.3 | 0.0 | 0.0 | 0.0 | 13.3 | 0.0 |
| TXx | 0.0 | 0.0 | 0.0 | 0.0 | 26.7 | 0.0 |
| TNn | 3.3 | 0.0 | 0.0 | 0.0 | 13.3 | 0.0 |
| TXn | 0.0 | 0.0 | 0.0 | 0.0 | 26.7 | 0.0 |
| DTR | 6.7 | 0.0 | 0.0 | 0.0 | 30.0 | 0.0 |
| WSDI | 0.0 | 0.0 | 0.0 | 0.0 | 26.7 | 0.0 |
| CSDI | 3.3 | 0.0 | 0.0 | 0.0 | 13.3 | 0.0 |
| **Precipitation indicators** | | | | | | |
| R95pTOT | 0.0 | 0.0 | 0.0 | 0.0 | 6.7 | 0.0 |
| R10MM | 0.0 | 0.0 | 0.0 | 0.0 | 6.7 | 0.0 |
| R20MM | 0.0 | 0.0 | 0.0 | 0.0 | 6.7 | 0.0 |
| RX1Daypcpn | 0.0 | 0.0 | 0.0 | 0.0 | 6.7 | 0.0 |
| RX5Daypcpn | 0.0 | 0.0 | 0.0 | 0.0 | 6.7 | 0.0 |
| CWD | 0.0 | 0.0 | 0.0 | 0.0 | 6.7 | 0.0 |
| CDD | 0.0 | 0.0 | 0.0 | 0.0 | 6.7 | 0.0 |
| PRCPTOT | 0.0 | 0.0 | 0.0 | 0.0 | 6.7 | 0.0 |
| SDII | 0.0 | 0.0 | 0.0 | 0.0 | 6.7 | 0.0 |
