# Supplementary material for: On people’s perceptions of climate change and its impacts in a hotspot of global warming
Source: PLoS One. 2025 Feb 13;20(2):e0317786. doi: 10.1371/journal.pone.0317786 (PMC11825050; doi:10.1371/journal.pone.0317786)
Supplement: S2 File — (DOCX) [file pone.0317786.s014.docx]

|  |  |  |  |
| --- | --- | --- | --- |


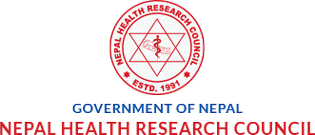


**;+s]t gDa/**

**Eco-bio-social drivers for effective *Aedes* vector prevention & control along a climatic gradient in Nepal - NAECO**

**A SURVEY INFORMATION AND GEO-REFERENCING**

| **qm=;+=** | **kl/0ffDo** |  |
| --- | --- | --- |
| A1 | Name of participant |  |
| A2 | Date of interview | Day  Month Year |
| A3 | Municipality |  |
| A4 | Cluster |  |
| A5 | Address |  |
| A6 | Latitude |  |
| A7 | Longitude |  |
| A8 | Height | ====================== |

**B= RESEARCH PARTICPANT INFORMATION**

| **S.N** | **kl/0ffDo** |
| --- | --- |
| B1 | Full name==================================================================================== |
| B2 | In what district and gaupalika/municipality were you born?  a= Distruct ========================= b= Rural municipality============== c Municipality===========================  d= Other country====================== |
| B3 | Age=============== in years |
| B4 | Sex Male Female |
| B5 | Which of the following ethnic groups you belong to?:  1 = Dalit 2 = Disadvantaged Janajatis 3 = Disadvantaged non Dalit Terai caste groups 4 = Religious minorities  5 = Relatively advantaged Janajatis 6 = Upper caste  99 = Does not know |
| B6 | Educational qualification: 1. Illiterate 2. Informal education 3. Primary 4. Secondary 5.Bachelor 6. Masters |
| B7 | Marital status: 1. Unmarried 2. Married 3.Divorced 4.Widow 5. Others……… |
| B8 | Occupation :  1. Agriculture 2. Business 3. Student 4.Service  5. Household work 6. Pensioned 7. Others…………….. |
| B9 | Are you part of the healthcare system as an affiliate or beneficiary?  1 = Yes 2 = No 3 = do not know |
| B10 | Are you affiliated to social insurance?  1 = Yes 2= No 3= Do not know |
| B11 | What is the monthly family income? (DO NOT READ OPTIONS, WAIT FOR ANSWERS)  1 = less than NRs. 10000 2 = NRS. 10,000-20,000  3 = 20,000-30,000 4 = 30,000-40,000  5 = 40,000-50,000 6 = more than 50,000  7 = do not know |
| B12 | Do you live by yourself? 1 = Yes 2 = No |
|  | Who is the final decision-maker in your home regarding the following decisions?  WAIT FOR RESPONSE (1 = a man; 2 = a woman; 3 = both; 4 = nobody; 5 = does not know)   1. Healthcare? 2. Healthcare of other members of the home? 3. Major purchases for the home? 4. Purchasing daily needs for the home? 5. Taking care of the house? |

**C=** **MIGRATION AND PERMANENCE**

| **S.N** | **kl/0ffDo** |
| --- | --- |
| C1 | For how long have you lived in this neighborhood? Days……… month……… year…….. |
| C2 | For how long have you lived in this house? Days……… month……… year……….. |
| C3 | Where did you live before living in this house? (DOES NOT READ OPTIONS, WAIT FOR ANSWERS)  1 = Same municipality,same zone 2 = Same municipality,different zone(urban/rural)  3 = Other municipality of this province 4 = Other province  5 = Other country 6 = do not answer |
| C4 | Where did you live when the earthquake in Nepal of 2015 occurred?  1= Chitwan 2 = Kathmandu 3 = Lalitpur 4 = Dhading  5 = Rasuwa 6 = Other district 7 = Other country  8 = Do not want to answer |

**D= CHARACTERISTICS OF THE HOUSING AND BASIC UTILITIES**

| **S.N** | **kl/0ffDo** | | |
| --- | --- | --- | --- |
| D1 | What type of housing is this?  1 = House 2 = Apartment 3 = Room(s) in tenancies 4 = Room(s) in other type of structure 5 = Other type of housing (tent, carp, wagon, ship, natural refuge, bridge, etc.) | | |
| D2 | What is the main use of your house?  1= Residence 2 = Business 3 = Restaurant  4 = Mix 5 = Other | | |
| D3 | How many floor are in your house?  If more than one, in which of them do you live? ……… | | |
| D4 | OBSERVE AND WRITE DOWN THE PREDOMINANT MATERIAL OF THE FLOOR OF THE HOUSE OCCUPIED BY THE HOME  1 = Marble, parquet, polished and lacquered wood; 2 = Carpet, wall to wall carpet; 3 = Tile, ceramic tile, vinyl, tablet, brick, polished non-lacquered wood;  4 = Rough wood, wood board or plank, other vegetal;  5 = Cement, gravel; 6 = Soil/sand | | |
| D5 | OBSERVE AND WRITE DOWN THE PREDOMINANT MATERIAL OF THE EXTERIOR WALLS OF THE HOUSE OCCUPIED BY THE HOME  1 = Brick or sight block 2 = Brick or reversed or plastered block;  3 = Stone, polished wood 4 = Footfall walls, adobe;  5 = Reversed bahareque 6 = Others | | |
| D6 | How many windows does your house have? | | |
|  | Windows have: |  |  |
|  | 1 = Glass, 1 = Yes 2 = No  2 = Net 1 = Yes 2 = No |  |  |
|  | 3 = Plastic 1 = Yes 2 = No  4 = Nothing 1 = Yes 2 = No  5 = Curtains 1 = Yes 2 = No  6 = Other 1 = Yes 2 = No |  |  |
| D7 | Which of these spaces does your house have? | | |
|  | 1 = Courtyard 1 = Yes; 2 = No |  |  |
|  | 2 = Lot or solar 1 = Yes; 2 = No |  |  |
|  | 3 = Front yard 1 = Yes, 2 = No  4 = Garage or parking spot 1 = Yes; 2 = No  5 = Rooftop or terrace 1 = Yes; 2 = No  6 = Green zones or common-property zones 1 = Yes ; 2 = No |  |  |
| D8 | If it has a backyard, how many trees taller than an adult are there? ……… | | |
| D9 | Show me your bathroom, OBSERVE AND WRITE DOWN WHAT KIND OF TOILET SERVICE DOES THE HOUSE HAVE?  1 = Toilet connected to sewerage 2 = toilet connected to septic tank  3 = Non-connected toilet 4 = Latrine (black hole, pit)  5 = Does not have any toilet | | |
| D10 | Which of the following public, private, or community utility services does your house have?  1= Power 1 = Yes 2 = No  2 = Natural gas connected to a public web? 1 = Yes 2 = No  3 = Aqueduct, water supply? 1 = Yes 2 = No  4 = Sewerage? 1 = Yes 2 = No  5 = Waste collection? 1 = Yes 2 = No | | |
| D11 | Do you have servant at home? 1 = Yes 2 = No | | |
| D12 | \| 1= Radio 1 = Yes 2 = No \| \| --- \| \| 2= Color TV 1 = Yes 2 = No \| \| 3 = Washing machine 1 = Yes 2 = No \| \| 4 = DVD 1 = Yes 2 = No \| \| 5 = Computer 1 = Yes 2 = No \| \| 6 = Working internet 1 = Yes 2 = No \| \| 7 = Air conditioning 1 = Yes 2 = No \| \| 8 = Fan 1 = Yes 2 = No \| | | |
| D13 | Which are the three sources of information you use more to know about what is going on every day in your country? Surveyor: DOES NOT READ THE ANSWERS, ONLY REPORT WHAT PEOPLE ANSWER SPONTANEOUSLY  1 = relatives, friends, and neighbors 2 = community informative  3 = local or community newspaper 4 = national newspaper  5 = radio 6 = television  7 = groups or associations 8 = work or business partners  9 = community leaders 10 = a government official  11 = NGOs 12 = internet | | |

**E. Effects of climate change in Nepal**

| **S.N** | **kl/0ffDo** | | | |
| --- | --- | --- | --- | --- |
| E1 | Current intensity of heat level in summer in comparison to last 5 to 10 yrs?  Very low low high very high same don’t know | | | |
| E2 | Present bitterness of cold in winter in comparison to last 5 to 10 years?  Very low low high very high same don’t know | | | |
| E3 | Change in rainfall timing in your areas compared to past 5 to 10 years?  Very low low high very high same don’t know | | | |
| E4 | Shifting of monsoon rainfall in your area earlier than past 5 to 10 years?  Very low low high very high same don’t know | | | |
| E5 | Prolonging of rainfall in post-monsoon and winter than past 5 to 10 years?  Very low low high very high same don’t know | | | |
| E6 | Experienced heavy rainfall in later years < | Yes | No | Don’t  No |
| **S.N** | **kl/0ffDo** | **Yes** | **No** | **Don’t**  **know** |
| E7 | Experienced drying of water resources in later years | 1 | 2 | 3 |
| E8 | Experienced increase in frequency of droughts in later years | 1 | 2 | 3 |
| E9 | Experience of mosquitoes in new areas or higher altitudes where not found before | 1 | 2 | 3 |
| E10 | Experienced the transmission of vector borne diseases new areas/higher altitudes | 1 | 2 | 3 |
| E11 | Experienced new diseases in humans in later years compared to past years | 1 | 2 | 3 |
| E12 | Experienced new diseases in your crops in later years compared to past years | 1 | 2 | 3 |
| E13 | Experienced new diseases in domestic animals in later years compared to past years | 1 | 2 | 3 |
| E14 | Experienced less snow in Himalayas in later years compared to past years | 1 | 2 | 3 |
| E15 | Experienced increasing of floods and landslides in later years compared to past | 1 | 2 | 3 |

**********************
