# Supplementary material for: On people’s perceptions of climate change and its impacts in a hotspot of global warming
Source: PLoS One. 2025 Feb 13;20(2):e0317786. doi: 10.1371/journal.pone.0317786 (PMC11825050; doi:10.1371/journal.pone.0317786)
Supplement: S3 File — (PDF) [file pone.0317786.s015.pdf]

Nepal Health Research Council, Kathmandu and Goethe University, Frankfurt am Main, Germany

Eco-bio-social drivers for effective *Aedes* vector prevention & control along a climatic gradient in Nepal - NAECO
(Focus Group Discussion Guidelines)

TAKING INFORMED CONSENT

After taking informed consent of participants, record the following socio-demographic characteristics of research participants participants will be recorded during introduction of research participants

Table: Socio-demographic characteristics of Focus Group Discussion Participants

S.N	Name of FGD participants	Address	Age (in years)	Sex	Marital status	Family size	Education level	Occupation	Annual income of family
1									
2									
3									
4									
5									
6									
7									
8									
9									
10									
11									
12									

Date: Time: Venue:

Introduction and greetings

Welcome to participants once again, highlight overview of discussion topic and make ground rules in a friendly and participatory way.

Main session

In order to focus the discussion, following topic/question will be asked:

1. Discussing about general environment and socio-economic condition of study area
2. Major environmental and climatic changes of study areas perceived/experienced by participants compared to past 5 to 10 years
3. Major changes in society such as settlement, migration and displacement, shifting in farming and land use, livelihood,
4. Household and caring burdens, urbanization, access to health care, public transportation, and provision of water supply, sanitation and hygiene
5. Information about mosquito-borne diseases dengue and chikungunya and their vectors
6. Sources of information and access of information (e.g., gender decision-making regarding health care of family members, household chores, maintenance and purchases),
7. Health care system (private vs public),
8. Family member's information about transmission of these diseases, their symptoms and treatment
9. Water availability, sources of water, water supply system
10. Quality, quantity and service of water supply
11. Water bill price and water service interruption,
12. Water storage behaviours and practices of people (definition of water storage, per type of water, per type of container, reasons for water storage),
13. Perceptions for water storage rationale by neighbors,
14. The relationship between water accumulation, perception of people about spatial and temporal distribution and abundance of mosquitoes, and
15. Perceived risks and health seeking behaviors including preventive measures from infection with dengue and chikungunya viruses.
16. Recommendations of research participants for prevention and control of mosquitoes and mosquito-borne diseases

Closing

Summarize discussion with confirmation, ask to supplement if anything has been missed, thank to participants and dismissal.

End

Nepal Health Research Council, Kathmandu and Goethe University, Frankfurt am Main, Germany

Eco-bio-social drivers for effective *Aedes* vector prevention & control along a climatic gradient in Nepal - NAECO

(Interview Guidelines)

After taking informed consent of interviewee, record the following socio-demographic characteristics of interviewee

Table: Socio-demographic characteristics of Focus Group Discussion Participant

S.N	Name of FGD participants	Address	Age (in years)	Sex	Marital status	Family size	Education level	Occupation	Annual income of family
1									

Date: Time: Venue:

Introduction and greetings

Welcome to interviewee once again, highlight overview of discussion topic.

Main session

In order to focus the discussion, following topic/question will be asked:

1. Discussing about general environment and socio-economic condition of study area
2. Major environmental and climatic changes of study areas perceived/experienced by participants compared to past 5 to 10 years
3. Major changes in society such as settlement, migration and displacement, shifting in farming and land use, livelihood,
4. Household and caring burdens, urbanization, access to health care, public transportation, and provision of water supply, sanitation and hygiene
5. Information about mosquito-borne diseases dengue and chikungunya and their vectors

6. Sources of information and access of information (e.g., gender decision-making regarding health care of family members, household chores, maintenance and purchases),
7. Health care system (private vs public),
8. Family member's information about transmission of these diseases, their symptoms and treatment
9. Water availability, sources of water, water supply system
10. Quality, quantity and service of water supply
11. Water bill price and water service interruption,
12. Water storage behaviours and practices of people (definition of water storage, per type of water, per type of container, reasons for water storage),
13. Perceptions for water storage rationale by neighbors,
14. The relationship between water accumulation, perception of people about spatial and temporal distribution and abundance of mosquitoes, and
15. Perceived risks and health seeking behaviors including preventive measures from infection with dengue and chikungunya viruses.
16. Recommendations of research participants for prevention and control of mosquitoes and mosquito-borne diseases

Closing

Summarize discussion with confirmation, ask to supplement if anything has been missed, thank to participants and dismissal.

End
