# Supplementary material for: On people’s perceptions of climate change and its impacts in a hotspot of global warming
Source: PLoS One. 2025 Feb 13;20(2):e0317786. doi: 10.1371/journal.pone.0317786 (PMC11825050; doi:10.1371/journal.pone.0317786)
Supplement: S4 File — (DOCX) [file pone.0317786.s016.docx]

# **S1 File. Detail results on climate extreme indices and social survey**.

### **Results: Climate extreme indices**

In Central Nepal, 12 climate extreme indices show a significant trend (11 in the Lowland, 18 in the Midland, 19 in the Highland) in a period of 37 years (1981-2017). Those climatic trends have intensified during the last 7 years (2011-2017) compared to the 30 years-period (1981-2010) as indicated by the significant change of 2 indices along the study gradient in Central Nepal (0 in the Lowland, 11 in the Midland, 1 in the Highland; Fig 3 and 4). Along the altitudinal gradient in Central Nepal, the climate parameters on an annual basis show an increase in the heat level in all regions (Past 37 years- Lowland: TNx, TN90p, Midland: TR, SU, TXx, TX90p, TN90p, Highland: SU, TX90p, TN90p; Past 7 years: Midland: TR, TN90p), a decrease in bitterness of cold next to the Lowland (Past 37 years: TX10p; Past 7 years: TNn, CSDI) especially in the Midland (Past 37 years- decrease in FD, TX10p, TN10p, CSDI, increase in TXn, TNn; Past 7 years- same as 37 years and significant DTR) and Highland (Past 37 years: decrease in TX10p, TN10p, CSCI, increase TNn, FD) while there was a decrease in heavy precipitation in all regions (Past 37 years: decrease in all regions in Rx5Day, R10mm, R20mm, Midland: especially CWD decreases and R95pTOT increases, Highland: SDII decreases; Figures S6 and S7,Figures S1, S2 and S8 Figs ). In summer and winter, the temperatures have dramatically increased, especially in the past 7 years in the Midland (summer: Past 37 years - Lowland: TNx, TX90p, TN90p; Midland: TR, SU, DTR, TNx, TXx, TX90p, TN90p; Highland: SU, TXx, TX90p, TN90p; Last 7 years: Midland: TR, TNx, TXx, TX90p, TN90p; Highland: TX90p; winter: Past 37 years – Lowland: increase TX10p, decrease TN10p; Midland: decrease FD, DTR,TX10p, TN10p, CSDI, increase: TXn, TNn; Past 7 years – Midland: as 37 years but TXn is not significant). In the Highlands, the winter temperatures also show a decreasing trend (decrease: DTR, TX10p, TN10p, CSDI; increase: TXN, TNn; Fig 3 and 4).

No overall consistent trend in the seasonal precipitation pattern is present, however in all altitudinal regions at least one weather variable increased either in the past 37 years or specifically the past 7 years in the pre-monsoon season (Past 37 years – Lowland: Rx5Day; Highland: CWD; Past 7 years: Midland: Rx5Day; Fig 4). Moreover, in the monsoon season, especially in the Midland and Highland some extreme precipitation events have increased (Past 37 years – Midland: Rx5Day increases, Highland: CWD, Rx5Day), while in the Midland one parameter decreased (Past 37 years: CWD). In the post-monsoon season, there were differences between the Lowland and Midland compared to the Highland (Past 37 years: Rx5Day increased in Lowland and Midland, Highland: decrease in Rx5Day, R10mm, Rx1Day). In winter, dryness was found to be increasing in the Lowland and Highland (Past 37 years- Lowland: increase CDD; Highland, decrease Rx1Day, Rx5Day; Fig 4, S3 Fig).

### **Detail discussion: Climate extreme indices**

Climate change has influenced the climate in Central Nepal over the last 37 years, in particular in the last 7-years (Figure 3). The warming effect of warm and cold temperatures is especially pronounced in the Mid- and Highland (Figure 3, S4 Fig). A direct comparison of Central Nepal with the whole country along the altitude (0-2655m amsl) shows consistently an increase of coldest/warmest days and coldest nights (TXx, TXn, TNn) and the percentage of warm days (TX90p) as well as a decrease in the percentage of cold days (TX10p; from 1986-2015; Poudel et al., 2020). Furthermore, Poudel et al., (2020) showed that the winter days in December and January in the Lowland (60-200m amsl) have become colder. Accordingly, in the Lowland of Central Nepal in winter cool days (TX10p) have increased while the nights (TN10p) have decreased. The overall warming effect in winter and summer is especially pronounced in the Midland of Nepal. The Midland area is monitored by a weather station in the capital Kathmandu and Lalitpur. Both cities lay directly next to each other on a plateau at approx. 1300m asl. Poudel (2020) describes this warming effect as a heat island effect, hence urbanization affects the temperature in the Midland (Karl et al., 1988; Maharjan and Regmi, 2015; Mishra et al., 2019; Mitchell, 1961; Poudel et al., 2020). Thus, the decreasing of greenery inside and outside the Kathmandu valley due to development of new urban and semi-urban areas is associated with an increase in the temperature of the valley (Midland; Mishra et al., 2019). Altitudinal region-specific differences between the Low-, Mid- and Highland are also associated with the varying level of climates that are prevalent in diversified topography and vegetation (Nath Paudel, 2016). The described climate analysis and other studies show that the indicators for climate changes in extreme temperatures in Nepal depend on the altitude as well as the latitude (Karki et al., 2020; Poudel et al., 2020; Thakuri et al., 2019).

Consistent seasonal trends over all regions in Central Nepal are not detected for the precipitation indices (Figure 4). High intensity-related precipitation extremes are increasing in the Lowland (seasonally: Rx5Day in pre-monsoon and post-monsoon), the Midland (annually: R95pTOT, seasonally: Rx5Day in monsoon and post-monsoon) and in the Highland (seasonally: Rx5Day increases in the monsoon, decreases in post-monsoon; Figure 4). These heavy precipitation extremes can lead to more floods in the study regions. Annually, the heavy precipitation decreased significantly (R10mm, R20mm, Rx5day) and a trend of increasing consecutive dry days (CDD) was observed in all study regions. Spatial variation of the precipitation in the whole country compared to Central Nepal also showed high differences between, and within, the seven provinces of Nepal (Sudurpashchim, Karnali, Lumbini, Gandaki, Bagmati, Madhesh and Province 1). In the provinces, altitudinal precipitation differences are present and precipitation is more intense in the mid-elevation areas (Sharma et al., 2020). Next to the extreme precipitation events (Sharma et al., 2020), an extension of the monsoon period can also lead to floods or landslides. In general, the monsoon period has extended in Nepal since 1981 (S9 Fig).

## **Results: Social survey**

### **Social data: Socio-demographic characteristics of the HHS participants in Central Nepal**

Out of a total of 660 households/individuals targeted for the study, 651 participants were enrolled for the questionnaire survey with a response rate of 98%. Among the total interviewed participants, 33.5% were resident of the Highland, 33.0% of the Midland and 33.5% from the Lowland, of all participants 64.2% were female and 35.8% male, while most of the research participants (46%) were between the ages of 30 to 44 years. Regarding the participants’ occupation, most of the participants in the Lowland (38.1%) and Midland (45.1%) were engaged in business, while most of the participants in the Highland (33.5%) were engaged in agriculture. All socio-demographic characteristics of the study participants were significantly different between the Lowland-Midland- and Highland (details in S5 Fig).

### **Perception on direct climate change impacts**

#### **Perception on summer and winter patterns**

The intensity of the current heat level during summer in comparison to the past 5 to 10 years was perceived as having increased by 62% of the HHS participants. Similarly, the majority of the HHS participants from Lowland (64.7%), Midland (75.3%), and Highland (45%) perceived an increased heat level in summer (P1). In agreement, both the FGD and IDI participants of the respective regions had experienced hotter summers. In Central Nepal, the present bitterness of cold during the winter in comparison to the past 5 to 10 years (P2) was perceived as having increased by 37.2% of the HHS participants (Fig 2). However, this strongly depended on the regional residential area of the participants. In the Lowland, there was a mixed HHS perception (feels same; 31.5%, decrease; 31.5%, increase; 30.6%) regarding the coldness of the winter, while in the Midland, the HHS participants (53.0%) perceived a decrease in winter cold, and in the Highland, the HHS participants (51.8%) perceived colder winter (P2 in Fig 2). Meanwhile, in the qualitative data, only the FGD and IDI participants of the Midland and Highland agreed with the HHS participants about the winter cold: a less cold winter in the Midland but colder winter in the Highland. The IDI and FGD participants from the Lowland perceived an increase of cold in the winter. In addition, the FGD and IDI participants from the Lowland were experienced more foggy days in winter. These perceptions are illustrated by the following quotes:

*"Yes, the days are becoming hotter in summer and colder in winter than before ",* IDI participant, Lowland.

*“Hotness has increased so much in summer if compared to the past. Before, we do not have to use fans but nowadays, we cannot stay without using a fan. Even, we are feeling less cold in winter nowadays.”* FGD participant, Midland.

*“Now the hotness during the summer and coldness during the winter have increased compared to the previous years.”* FGD participant, Highland.

#### **Perception on monsoon patterns**

The majority of the HHS, FGD and IDI participants of the Lowland, Midland and Highland perceived a change in the rainfall timing. The rainfall timing was perceived to have “much changed” by 61. 4% and “little changed” by 13.1% of the HHS participants (P3). The monsoon rainfall was perceived to have shifted to an earlier date by HHS participants (51.9%) during the past 5 to 10 years (P4). This response of the HHS participants concerning an earlier onset of the monsoon is common in the Lowland, Midland and Highland (Fig 2). However, the IDI and FGD participants showed disagreement based on their residential area, on the monsoon timing: the Highland participants perceived an earlier onset of the monsoon season, whereas the Midland participants perceived both an earlier and delayed onset of monsoon, while the Lowland participants perceived a delay in the onset of monsoon rainfall (P3, P4, P5). The rainfall season during the post-monsoon and winter season was perceived by 60% of the HHS participants as being prolonged when compared to the past 5 to 10 years. This HHS response is common in the Lowland, Midland and Highland. The FGD and IDI participants of the Lowland and Midland perceived a prolonging of the monsoon in the post-monsoon and winter season, while the FDG and IDI participants of the Highland showed a disagreement (P5). A more heavy rainfall in recent years, as compared to the past, was perceived by 71.7% of the HHS participants (Fig 2), together with the FGD and IDI participants (P6).

*I think the rainfall pattern has changed. Nowadays the monsoon starts lately and continue for a long period. I have felt the shifting of monsoon as well as the shifting in fruit bearing time of the seasonal fruits like Kaphal (Bayberry), Aiselu (Raspberry).”,* IDI participant, Lowland.

*"Normally, the monsoon supposed to start from the month of Jestha (May/June) or Ashar (June/July) but nowadays, sometimes it starts lately and sometimes earlier. There is heavy rainfall this year. The rainfall started earlier this year and continue until the long period.”* FGD participant, Midland.

### **Perception on environmental change and indirect climate change impacts**

#### **Environmental change**

The FGD and IDIs participants have experienced common environmental changes such as the conversion of agricultural land and forest areas into settlement or industrial areas. Moreover, the participants have experienced an increase in environmental pollution (air, water, soil and noise), poor waste management and the drying-up of water sources. They reported rapid population growth as the main cause of environmental chance, beside land use changes and infrastructural development, as illustrated by the following quote:

*"Previously almost all the people used to depend on agriculture for their livelihood. However, the population of this district has rapidly increased nowadays due to modernization. As a result, the fertile land used for agriculture were now occupied by industries, factories and buildings.”* IDI-male expert, Lowland

#### **Disasters**

The participants from the study regions in Central Nepal reported on the direct and indirect climate-induced disasters. The strongest perceptions of disasters were derived from the Highland participants. The IDI participants (Lowland, Midland and Highland) reported an increasing trend of heavy rainfall, landslides, floods and soil erosion from all the altitudinal regions. They also reported that the disasters affected public life and public properties, including roads and water supply systems.

*"Due to heavy rainfall, we are experiencing the natural disasters like flood, landslide, and soil erosion too. This heavy rainfall uses to block the roads as well.”* IDI participant, Lowland.

The perceptions on increasing floods and landslides differ between the HHS participants residing in the Lowland, Midland and Highland (p<0.001; P15 in Fig 5). Their response was higher if residing in the Highland (53.2%). Similarly, the majority of participants (58.8%) perceived the drying-up of water resources in recent years (P7). This response was higher in the Midland (80%) than in the Lowland and Highland (Fig 5). The FGD and IDI participants had also witnessed the drying-up of water resources in the Lowland, Midland and Highland areas.

“*The water sources have dried up nowadays, that is why we are facing the scarcity of water.*” IDI participant, Midland.

#### **Land use and agricultural change**

With regard to land-use, IDI and FGD participants perceived that the involvement of people in agriculture had declined in recent years, although most of the people still depended on agriculture. They stated that the conversion of fertile, agricultural land into settlement areas as well as industrial regions had reduced the areas of agricultural land. The HHS and IDI participants from all regions (Lowland, Midland, Highland) reported that unusual rainfall patterns and climate-induced disasters had adversely affected the production of food grains, vegetables and fruits. They also reported people’s increasing dependency towards markets.

*"Fertile lands are now changing to residential areas. People are making houses at fertile lands and we are depending on markets for foods.”* FGD participant, Lowland.

The majority of the HHS participants residing in the Lowland (48.1%), Midland (45.1%) and Highland areas (42.3%) reported new crop diseases in recent years (P12 in Fig 5). Correspondingly, the IDI and FGD participants reported that the presence of pests and insects in vegetables and crops had adversely affected their livelihoods. This eventually, had caused an increase in the use of chemical pesticides in agricultural farming, as illustrated by the following quote.

*"Yes, nowadays the vegetables are damaged by pests and insects. More than half amount of vegetables has damaged because of insects. We have to use pesticides to kill the insects.*” FGD participant, Highland.

#### **Biodiversity**

The FGD and IDI participants perceived both negative and positive impacts of both climate change and environmental change on the distribution patterns of both plant and animal species. The FGD participants reported the shifting of plant species to new altitudinal ranges, the disappearance of plant species from existing areas and a change in the timing of the phonological events of plants. In addition, they also reported the new occurrence of poisonous snakes in the Highland areas in recent years when compared to the past, as illustrated by the following quotes:

*“Now, we can grow the mango trees here. Before, the mango’s trees did not used to grow here, but now the mangos are grown here which is also an indication of increasing temperature.*”, FGD participant, Highland.

“*Before, we could find so many oranges here, but now we can’t find them. Most of the orange trees have died nowadays. In the month of Magh (January/February), we could see the rhododendron flowers in our villages, but now we cannot see them in the same month as before. Nowadays, they use to flower lately.”* FGD participant, Midland.

*“In hilly areas, we didn’t encountered Cobra, Karet (common Krait) species of snake before, but now we can see them. Due to increasing temperature, these types of snakes are able to adjust there in the hilly areas too.”* IDI participant, Lowland.

#### **Human health and disease pattern**

Climate-induced changes of vector-borne diseases were perceived by the majority of participants (HHS, IDI, FGDs) across the three altitudinal regions. The FGD and IDI participants reported that the distribution of the vectors due to climate change were now becoming a problem throughout the year in the Lowland and Midland areas, as well as the spreading of vector populations to the Highland has been observed. In accordance, most of the HHS participants (68.3%) had witnessed vectors (mosquitoes) in new areas where they had not seen the vectors before (P9 in Fig 5). In the Highland, the perception of mosquitoes in the new areas was stronger than in the Lowland and Midland (p<0.001). Instead, more participants from the Lowland (38.9%) and Midland (42.8%) experienced the transmission of the vector-borne diseases in new areas when compared to those residing in the Highland (33.6%) (P10 in Fig 5).

“*Kathmandu was a cold city in the past so the number of mosquitoes were also less in previous years. However, their population has increased nowadays and they use to stay for long time. I have never heard about dengue cases from Kathmandu before, but nowadays, dengue cases has reported.”,* IDI participant (hospital-based medical expert), Midland.

Few HHS participants from the Lowland (19%), Midland (20%) and Highland (8.6%) reported new human diseases in recent years when compared to the past 5 to ten years (P11 in Fig 5). However, the FGD and IDI participants experienced the change in the pattern of both communicable as well as non-communicable diseases due to environmental and climate change. They witnessed the emergence of vector-borne diseases such as dengue and chikungunya in the three study regions. Seasonal flu was another problem reported by the IDI and FGD of the three study regions. According to them (IDI, FGD), the seasonal flu has spread very fast in recent years but now also presents in a more severe form. The common cold and cough used to be a normal health problem which could be treated by normal remedies such as hot water, ginger water and salt water. However, nowadays the cold has become a more severe form that takes a longer time for recovery. Both the IDIs and FGD participants of the three regions reported increasing cases of non-communicable diseases such as diabetes and blood pressure (Fig 5). In addition to the climate-induced health burden, the multifactorial causes of the increasing cases of non-communicable diseases were well perceived by the participants. The IDI and FGD participants perceived food habits, the lifestyles of people, culture, environmental pollution, and the use of pesticides in crops, vegetables and fruits as potential risk factors for the increasing trend of non-communicable diseases.
