# Supplementary material for: On people’s perceptions of climate change and its impacts in a hotspot of global warming
Source: PLoS One. 2025 Feb 13;20(2):e0317786. doi: 10.1371/journal.pone.0317786 (PMC11825050; doi:10.1371/journal.pone.0317786)
Supplement: Attachment — Submitted filename: renamed_b78fa.docx [file pone.0317786.s017.docx]

**Review Comments on "On People’s Perceptions of Climate Change and Its Impacts in a Hotspot of Global"**

- The paper presents valuable insights into people's perceptions of climate change in a critical area. The introduction effectively sets the context and highlights the importance of understanding local perspectives.
- Overall, the abstract is well-written. However, it would be beneficial to mention the different altitudinal regions covered in the study. Including a line on policy recommendations or how this study can inform climate change adaptation strategies for mountain communities would enhance its impact.
- The introduction is generally well-structured. It could be strengthened by integrating some reports from ICIMOD, which have conducted extensive work related to climate change and disaster risk assessment in Nepal.
- The methodology is generally well-organized, but further clarification is needed on the following points:
  - **Sample Selection:** More detail on how participants were chosen would enhance the study's credibility.
  - **Data Collection Methods:** Elaborating on the tools used (e.g., surveys, interviews) and any validation processes undertaken would be beneficial.
  - **Statistical Analysis:** A more thorough explanation of the analytical techniques used would improve the transparency of the results.
  - Please add a few lines regarding household consent and ethical approval processes.
- Including a map of the study area and the geographic locations of data collection would be a valuable addition.
- Clarify the consent obtained from the relevant department for data usage. Specifically, detail the procedure for downloading data from their website (e.g., the withdrawal of the monsoon and monsoon season length obtained from the Department of Hydrology and Meteorology, Kathmandu, Nepal).
- The results are clearly presented. However, consider providing more contextual analysis that links the results to existing literature, which would strengthen the argument and illustrate how findings align or contrast with previous studies.
- Some discussion points could benefit from deeper exploration, particularly concerning the implications of the findings on local policy and community actions. It would be helpful to mention whether climate-induced disaster risk has increased or decreased and how community perceptions relate to crop production changes.
- If possible, please provide the statistical values (p-values) regarding community perceptions of climate change, as mentioned in the data. It would also be useful to present p-values for lowland, midland, and highland areas in a table titled "People's Perceptions on Climate Change."
- The conclusion summarizes the main findings well, but it would be beneficial to include more specific recommendations for stakeholders based on the study's findings. Additionally, highlighting the limitations of the study and suggesting areas for future research would provide a more balanced view.
- References: Ensure all references are up-to-date and relevant, particularly in the context of climate change literature. This will enhance the paper's credibility and scholarly contribution.
