# Supplementary material for: On people’s perceptions of climate change and its impacts in a hotspot of global warming
Source: PLoS One. 2025 Feb 13;20(2):e0317786. doi: 10.1371/journal.pone.0317786 (PMC11825050; doi:10.1371/journal.pone.0317786)
Supplement: Attachment — Submitted filename: PlosOne2024_2.docx [file pone.0317786.s018.docx]

**Review: PONE-D-24-46221**

**Title:** On people’s perceptions of climate change and its impacts in a hotspot of global

warming

1. The paper offers valuable insights into peoples’ perceptions of climate change in selected sites of Nepal systematically compares those perceptions with climate indicators derived from meteorological data. Hence the study investigates specific socio-economic and other contexts (differed across the altitudinal regions) shaping peoples’ perceptions and provide useful recommendation for adaption and mitigation measures.

Abstract

**Introduction:**

2. The problem statement is too generalized “None of the studies incorporated a comprehensive set of climate indicators along with people's perceptions [16,29–36]. I suggest providing some specific examples of shortcomings from the previous studies that motivates the current paper to adopt certain approach (coupling objective climate indicators with subjective peoples’ perceptions) for the study to fill the gaps.

**Data and Methods:**

3. Comprehensively written and systematically elaborated. Need to mention, to what extent the climate data used in the study was received from the weather stations locating in the proximity of the sites selected for household surveys and interviews.
4. Information about study sites/regions is missing, which need to be given under a sub-heading in the Material and Methods section. A map of the study sites may also be useful for the readers. Although it is very briefly mentioned in the introduction section but understanding the socio-ecological and other features of the study sites will be useful for the reader.

**Results and discussions**

5. Impacts of climate change on human health not been sufficiently reflected in the results as it is done in discussion section such as distribution of venomous snakes, communicable (influenza) and non-communicable diseases (diabetes, blood pressure).
6. A conclusion and recommendation section are missing towards the end of the Discussion. Although it recommends certain measures for adaptation and recommendations. The study has could be useful to explore peoples’ perceptions of climate change *viz-a-viz* weather data in similar geographical landscapes, therefore

General

7. Typos need to be corrected, e.g. line 182.
