# Supplementary material for: On people’s perceptions of climate change and its impacts in a hotspot of global warming
Source: PLoS One. 2025 Feb 13;20(2):e0317786. doi: 10.1371/journal.pone.0317786 (PMC11825050; doi:10.1371/journal.pone.0317786)
Supplement: Attachment — Submitted filename: Response to Reviewers.docx [file pone.0317786.s019.docx]

16^th^ December 2024

Tasawar Baig, PhD
Academic Editor
PLOS ONE

Re: PONE-D-24-46221R1 “On people’s perceptions of climate change and its impacts in a hotspot of global warming”

Dear Dr. Baig,

Thank you very much for your email and for your comments on our manuscript. We have carefully revised the manuscript in response to the extensive and insightful comments we received from you and two reviewers. Please find enclosed our revised manuscript “On people’s perception of climate change and its impacts in a hotspot of global warming”, which we are submitting as an original article for consideration and publication in **‘PLOS ONE’.**

In particular, the reviewers requested more clarity in the methodology part and provided their valuable suggestions to improve the manuscript. Therefore, we have now revised the text and added more information according to the reviewers' suggestions. Appended below is the list of all reviewer comments, along with our responses to each point. We have also highlighted the changes in the marked-up copy of the manuscript.

Additionally, we have updated the manuscript according to Journal requirements and included a complete copy of PLOS’ questionnaire on inclusivity in global research in our revised manuscript as “S1 file”. Additionally, we have also included the household questionnaire and interview guidelines (Focus group discussions and in-depth interviews) as supportive files “S2 file” and “S3 file” respectively in the revised submission. As a result, the previous file S1 has been renamed as S4. Similarly, we have added Fig. 1 (Map of the study area) in the revised manuscript as per the reviewer’s comment. Therefore, the previous figures Fig. 1, Fig. 2, Fig. 3, and Fig. 4 have been renumbered as Fig. 2, Fig. 3, Fig. 4, and Fig. 5, respectively.

We have also updated the financial disclosure “The work was funded by the Federal Ministry of Education and Research of Germany (BMBF) under the project AECO (Number 01Kl1717) as part of the National Research Network on Zoonotic Infectious Diseases of Germany. The funders had no role in study design, data collection and analysis, decision to publish, or preparation of the manuscript” in the cover letter as per your suggestions. We have also updated and reviewed the references list to ensure if they complete and correct.

We are very hopeful that this revised version will be suitable for publication in PLOS.

Yours sincerely,

Parbati Phuyal & Isabelle Kramer (on behalf of all authors)

Journal requirements:

When submitting your revision, we need you to address these additional requirements.

1.  Please ensure that your manuscript meets PLOS ONE's style requirements, including those for file naming. The PLOS ONE style templates can be found at

[https://journals.plos.org/plosone/s/file?id=wjVg/PLOSOne_formatting_sample_main_body.pdf](https://track.editorialmanager.com/CL0/https:%2F%2Fjournals.plos.org%2Fplosone%2Fs%2Ffile%3Fid=wjVg%2FPLOSOne_formatting_sample_main_body.pdf/1/010f019326cc716a-91eac322-12e7-442c-a5ba-9665e5b1d872-000000/1NrBcuyz6z_zEu0yIKDwG-UTDd8YsphFfkw4koUiCSg=185)  and

[https://journals.plos.org/plosone/s/file?id=ba62/PLOSOne_formatting_sample_title_authors_affiliations.pdf](https://track.editorialmanager.com/CL0/https:%2F%2Fjournals.plos.org%2Fplosone%2Fs%2Ffile%3Fid=ba62%2FPLOSOne_formatting_sample_title_authors_affiliations.pdf/1/010f019326cc716a-91eac322-12e7-442c-a5ba-9665e5b1d872-000000/S-QWVe-xylq3Lj1-uj0rrDiJgZA9BG1SLfGLfRNjias=185)

\## Thank you for your suggestions and we have carefully done formatting as suggested.

2. Please include a complete copy of PLOS’ questionnaire on inclusivity in global research in your revised manuscript. Our policy for research in this area aims to improve transparency in the reporting of research performed outside of researchers’ own country or community. The policy applies to researchers who have travelled to a different country to conduct research, research with Indigenous populations or their lands, and research on cultural artefacts. The questionnaire can also be requested at the journal’s discretion for any other submissions, even if these conditions are not met.  Please find more information on the policy and a link to download a blank copy of the questionnaire here: <https://journals.plos.org/plosone/s/best-practices-in-research-reporting>. Please upload a completed version of your questionnaire as Supporting Information when you resubmit your manuscript.

\## We have included the copy of PLOS questionnaire on inclusivity in global research in the revised manuscript under the section Materials and methods, Manuscript line: 122-124, also included the questionnaire as S1 checklist file and provided the link accordingly as per the journal requirement.

3. Thank you for stating the following financial disclosure:

“The work was funded by the Federal Ministry of Education and Research of Germany (BMBF) under the project AECO (Number 01Kl1717) as part of the National Research Network on Zoonotic Infectious Diseases of Germany.”

Please state what role the funders took in the study.  If the funders had no role, please state: "The funders had no role in study design, data collection and analysis, decision to publish, or preparation of the manuscript."

If this statement is not correct you must amend it as needed.

Please include this amended Role of Funder statement in your cover letter; we will change the online submission form on your behalf.

\## We have added a detailed financial statement (“The work was funded by the Federal Ministry of Education and Research of Germany (BMBF) under the project AECO (Number 01Kl1717) as part of the National Research Network on Zoonotic Infectious Diseases of Germany. The funders had no role in study design, data collection and analysis, decision to publish, or preparation of the manuscript”) in the cover letter as per your suggestion.

4. Please review your reference list to ensure that it is complete and correct. If you have cited papers that have been retracted, please include the rationale for doing so in the manuscript text, or remove these references and replace them with relevant current references. Any changes to the reference list should be mentioned in the rebuttal letter that accompanies your revised manuscript. If you need to cite a retracted article, indicate the article’s retracted status in the References list and also include a citation and full reference for the retraction notice.

\## We haven’t cited any retracted paper. However, we have updated and reviewed the references as per your suggestions.

**************


**Reviewer’s comments:**

**Reviewer-1 Comments:**

Comment 1: The paper presents valuable insights into people's perceptions of climate change in a critical area. The introduction effectively sets the context and highlights the importance of understanding local perspectives.

\## Thank you so much for your comments and appreciation.

Comment 2: Overall, the abstract is well-written. However, it would be beneficial to mention the different altitudinal regions covered in the study. Including a line on policy recommendations or how this study can inform climate change adaptation strategies for mountain communities would enhance its impact.

\## Thank you so much for the comments. We have done as suggested. We have also rephrased some sentences for more clarity. New Manuscript lines: 22-30 and 33-42.

Comment 3: The introduction is generally well-structured. It could be strengthened by integrating some reports from ICIMOD, which have conducted extensive work related to climate change and disaster risk assessment in Nepal.

Response 3: Thank you so much for your suggestions. We have added the reports from ICIMOD as per your suggestions. Manuscript lines: 60-62

Comment 4: The methodology is generally well-organized, but further clarification is needed on the following points:

- **Sample Selection:** More detail on how participants were chosen would enhance the study's credibility.
- **Data Collection Methods:** Elaborating on the tools used (e.g., surveys, interviews) and any validation processes undertaken would be beneficial.
  - **Statistical Analysis:** A more thorough explanation of the analytical techniques used would improve the transparency of the results.

**##** Thank you so much for the suggestions. Participants from the selected household were selected randomly. All detailed information regarding sampling collection, data collection methods and statistical analysis was already included in the manuscript. However, we have now updated more information as per your suggestions, Manuscript lines: 185-236. In addition, we have now provided the sub headings by including details accordingly for more clarification.

i) Details about quantitative data collection methods and sample selection could be found under the new heading **Quantitative data collection methods and sampling strategy**, Manuscript line: 186
ii) Details about statistical analysis could be found under the new heading **Quantitative data analysis,** Manuscript line: 208
iii) Details about qualitative data collection methods and sample selection could be found under the new heading **Qualitative data collection methods and sampling strategy, Manuscript line: 215** and data analysis methods under the heading **Qualitative data analysis method, Manuscript line: 224/ 224-236**

Comment 5: Please add a few lines regarding household consent and ethical approval processes.

\## Thank you for the suggestions. The information about household consent and ethical approval process is provided under heading Ethical approval. Manuscript lines: 114-121

Comment 6: Including a map of the study area and the geographic locations of data collection would be a valuable addition.

\## Thank you; we have included a map of the study area and geographical locations of data collection sites in Fig 1.

Comment 7: Clarify the consent obtained from the relevant department for data usage. Specifically, detail the procedure for downloading data from their website (e.g., the withdrawal of the monsoon and monsoon season length obtained from the Department of Hydrology and Meteorology, Kathmandu, Nepal).

Response 7: Thank you. Regarding the meteorological data, we have obtained the meteorological data and information from the Department of Hydrology and Meteorology, Kathmandu Nepal as per their institutional policy. We have also duly acknowledged the Department of Hydrology and Meteorology, Kathmandu, Nepal for providing meteorological data and information to meet their requirements.

Comment 8: The results are clearly presented. However, consider providing more contextual analysis that links the results to existing literature, which would strengthen the argument and illustrate how findings align or contrast with previous studies.

\## Thank you so much for your suggestions. We have already linked our findings to existing literature by conducting a detailed literature analysis and summarized data from 17 different studies (including multiple studies from HKH and other mountainous regions: Andes + Alps) in a table format in Supplement Table S1 to compare results to our dataset. However, we have now rephrased the sentences and updated the links where needed by showing our findings and links to the results to strengthen the argument as per your suggestions. Manuscript lines: In overall discussion section

Comment 9: Some discussion points could benefit from deeper exploration, particularly concerning the implications of the findings on local policy and community actions. It would be helpful to mention whether climate-induced disaster risk has increased or decreased and how community perceptions relate to crop production changes.

\## Thank you so much for the suggestions. We have now updated the information as per your suggestion. New manuscript line: 444-447. We have also updated information regarding community perception relate to crop production. Manuscript line: 452-460/ Manuscript lines: 383-386. The detail findings from social survey (HHs, IDI, FGD) is also provided in the supplementary file S4.

Comment 11: If possible, please provide the statistical values (p-values) regarding community perceptions of climate change, as mentioned in the data. It would also be useful to present p-values for lowland, midland, and highland areas in a table titled "People's Perceptions on Climate Change."

\## Thank you so much for your suggestions. We added in the table caption of Table 1 more details on what is exactly given to clarify what we exactly summarize in the Table. In general, the p-values regarding community perceptions of climate change, P1- P6 are provided in the figure named Fig 2 on the left hand side. Also in all other perception figures p-values are present.

Comment 12: The conclusion summarizes the main findings well, but it would be beneficial to include more specific recommendations for stakeholders based on the study's findings. Additionally, highlighting the limitations of the study and suggesting areas for future research would provide a more balanced view.

\## Thank you. We have now updated the conclusion and recommendations section and provided specific recommendations for stakeholders based on the study’s findings as per your suggestions. Manuscript lines: 491-533. We have also updated the limitations of the study as per your suggestions. Manuscript line: 547-552

Comment 13: References: Ensure all references are up-to-date and relevant, particularly in the context of climate change literature. This will enhance the paper's credibility and scholarly contribution.

Response 13: Thank you so much for your suggestions. We have carefully checked and updated as per your suggestions.

****************

**Reviewer- 2 Comments**

Comment 1: The paper offers valuable insights into peoples’ perceptions of climate change in selected sites of Nepal systematically compares those perceptions with climate indicators derived from meteorological data. Hence the study investigates specific socio-economic and other contexts (differed across the altitudinal regions) shaping peoples’ perceptions and provide useful recommendation for adaption and mitigation measures.

\## Thank you so much.

**Abstract**

**Introduction:**

Comment 2: The problem statement is too generalized “None of the studies incorporated a comprehensive set of climate indicators along with people's perceptions [16,29–36]. I suggest providing some specific examples of shortcomings from the previous studies that motivates the current paper to adopt certain approach (coupling objective climate indicators with subjective peoples’ perceptions) for the study to fill the gaps.

\## Thank you so much for your suggestions. As per your suggestion, we have added an explanation on what specifically is the shortcoming in regard to the climatic data. Manuscript line: 78-80/ 86-88. In general, we have already provided shortcomings from the previous studies with examples in the manuscript lines, 74-77. In addition, a comparison table is already present with 17 previous studies described in Supplement table S1. We have added the link to the file in the manuscript line: 80 that highlights the summary of the literature.

**Data and Methods:**

Comment 3: Comprehensively written and systematically elaborated. Need to mention, to what extent the climate data used in the study was received from the weather stations locating in the proximity of the sites selected for household surveys and interviews.

\## Thank you. The details about the approximate distance between social survey sites, and weather stations, altitudes of survey sites and weather stations were already present inside the file name supplementary Tables S2-S3 under “table S1”. However we have now provided the link in the manuscript line; 165-166.

Comment 4: Information about study sites/regions is missing, which need to be given under a sub-heading in the Material and Methods section. A map of the study sites may also be useful for the readers. Although it is very briefly mentioned in the introduction section but understanding the socio-ecological and other features of the study sites will be useful for the reader.

\## Thank you so much for your suggestions. We have now added the subheadings **Study Area** and provided additional information of the study sites as per your suggestions. Manuscript lines: 134/ 144-148. We have also added a map of the study sites (districts) as Fig1.

**Results and discussions**

Comment 5: Impacts of climate change on human health not been sufficiently reflected in the results as it is done in discussion section such as distribution of venomous snakes, communicable (influenza) and non-communicable diseases (diabetes, blood pressure).

\## Thank you. Earlier, the detailed findings were provided in the S4 file. However, we have now provided the detail findings regarding the distribution of venomous snakes, communicable and non-communicable diseases in the result section as per your suggestions. Manuscript lines: 373-382

Comment 6: A conclusion and recommendation section are missing towards the end of the Discussion. Although it recommends certain measures for adaptation and recommendations. The study has could be useful to explore peoples’ perceptions of climate change *viz-a-viz* weather data in similar geographical landscapes, therefore

\## Thank you so much for your feedback and suggestions. We have now added a conclusion and recommendations part with heading. Hence, we rearrange the existing information and provide additional information to make it more clear and structural. Manuscript lines: 490-532

General

Comment 7: Typos need to be corrected, e.g. line 182.

\## Thank you. We have corrected as per your suggestion.

************
